# Detection of Cell Carcinogenic Transformation by a Quadruplex DNA Binding Fluorescent Probe

**DOI:** 10.1371/journal.pone.0086143

**Published:** 2014-01-28

**Authors:** Tsung-Lin Yang, Lin Lin, Pei-Jen Lou, Ta-Chau Chang, Tai-Horng Young

**Affiliations:** 1 Department of Otolaryngology, National Taiwan University Hospital and College of Medicine, Taipei, Taiwan; 2 Research Center for Developmental Biology and Regenerative Medicine, National Taiwan University, Taipei, Taiwan; 3 Institute of Biomedical Engineering, College of Medicine and College of Engineering, National Taiwan University, Taipei, Taiwan; 4 Institute of Atomic and Molecular Sciences, Academia Sinica, Taipei, Taiwan; Glasgow University, United Kingdom

## Abstract

Cancer can be easily treated when found early. A probe capable of detecting cell transformation may increase the success rate of early diagnosis of cancer. In this report we have tested the ability of a fluorescent, quadruplex DNA binding probe, 3,6-bis(1-methyl-4- vinylpyridinium) carbazole diiodide (BMVC), to detect cell transformation in vitro. BMVC was applied to living cells in several different models of cell transformation, and the fluorescence signals of BMVC were measured. The degrees of cell transformation in these models were characterized by alterations in cellular morphological phenotype and subcellular organization. When BMVC probes were applied, the number of BMVC-positive cells increased in accordance with the degree of transformation. BMVC was capable of significantly detecting formation of foci, increased cellular motility, cell proliferation, cell apoptosis, anchorage-independent growth, and increased invasiveness of transformed cells. These results demonstrate the ability of BMVC probes to detect cell transformation and indicate that BMVC is of promise for use as a probe in early cancer detection.

## Introduction

Cancer can be easily treated when found early. Regardless of advances in treatment modalities, the early detection of cancer still remains a challenge [1]. Carcinogenesis is a multistep and multifocal process involving clonal expansion and spreading of transformed cells [2]–[6]. Clinically, the number of patients having precancerous lesions is far more than those with malignant tumors. Accurate prognostication of patients with premalignant lesions may prevent them from becoming serious cancerous illness [7]–[9]. Clinically, the standard method of identifying precancerous lesions is based on the pathological examinations requiring multi-step procedures and qualified pathologists. To develop more convenient and efficient methods, several carcinogenic biomarkers have been investigated during the past decades [1], [10]–[15]. However, the complicated and labor-intensive procedures render these techniques far away from routine use [16].

3,6-bis(1-methyl-4-vinylpyridinium) carbazole diiodide (BMVC) is a molecule made of carbazole derivatives [17]–[19]. BMVC displays a preferential binding to the G-quadruplex structure of DNA, and its intensity of fluorescence increases during binding reactions [17]–[19]. A BMVC probe can be used to differentiate cancer cells from normal cells [18]. Thus, using a simple handheld device, an acceptable diagnostic accuracy of cancer cells can be instantly achieved, even for a non-specialist [20], [21]. The major advantages of BMVC probes are mainly based on two distinct properties of this fluorescence probe: a significant increase of the fluorescence yield upon interaction with DNA, and the large time lag of adhesion of BMVC to the nucleus between cancer cells and normal cells [21].

Since BMVC can be used to differentiate cancer cells from normal cells, it warrants further investigation of its applications of detecting premalignant lesions. In this study, we explore the capacity of BMVC probes for detecting cell behaviors during carcinogenic transformation. BMVC probes were applied in several well-recognized cell transformation models [22]–[26]. In these inducible models, the degree and the process of malignant transformation of cells can be monitored, which is helpful for elucidating the capacities of BMVC probes. These results provide evidence of the capacities of BMVC probes to be developed into an agent of sensing cell transformation, which is of great potential for early cancer detection and screening.

## Materials and Methods

### BMVC synthesis and testing

We synthesized 3,6-bis(1-methyl-4-vinylpyridinium iodine) carbazole (BMVC) according to the procedure described previously [27]. Briefly, 3,6-dibromocarbazole (1.63 g, 5 mmole, Sigma-Aldrich, St. Louis, MO, USA) and the mixture of palladium(II) acetate (15 mg, Strem) and tri-o-tolyl phosphine (150 mg, Sigma-Aldrich, St. Louis, MO, USA) were added to a high pressure bottle. This mixture was subsequently mixed with the solvent pair (triethylamine 5 mL/tetrahydrofuran 15 ml) and 4-vinylpyridine (2 g, 20 mmole, Merck). The bottle was sealed after bubbling with nitrogen for 10 minutes. The system was kept under 105°C for three days, and the precipitant was collected and extracted with H_2_O/CH_2_Cl_2_ twice. The filtered insoluble solid was dissolved in tetrahydrofuran, and then dried by MgSO_4_. The product, 3,6-di(4-vinylpyridine) carbazole, was collected by recrystallization from tetrahydrofuran filtrate [28]. In the preparation of BMVC probes, BMVC stock solution was dissolved in dimethyl sulfoxide (DMSO) at 2 mg/ml, which was further diluted to a working concentration of 2 μM when preparing the BMVC probes. In BMVC testing, cells growing on 6 cm culture dishes were treated with 2 μM BMVC for 15 minutes in a 5% CO_2_ incubator at 37°C, and then washed thoroughly. The signal of BMVC was detected and analyzed using fluorescence microscopy. BMVC fractions meant the fraction of cells staining positively with BMVC in the biological assays.

### Cell culture

Mouse fibroblast cell lines (BALB/c 3T3, clone A31-1-1) were obtained from the American Type Culture Collection (ATCC). Cell culture was performed based on the protocol suggested by ATCC, and maintained in an incubator with 37°C, 5%CO_2_, and 95% humidity. Cell number was decided by trypan blue staining and a hemacytometer.

### MCA-induced cell transformation assays (CTA)

To investigate the capacity of BMVC for detecting cell transformation during carcinogenesis, a system of inducible cell transformation is important for *in vitro* analysis [28]–[30]. A BALB/c 3T3 cell transformation assay was performed according to a modified protocol as previously described [22]–[26]. Cells were seeded at a density of 10^4^ cells/60 mm dish and then incubated for 24 hours. Subsequently, the cultured cells were treated with different doses of 3-methyl-cholanthrene (MCA, Sigma-Aldrich, St. Louis, MO, USA). Untreated BALB/c 3T3 cells and those treated only with solvent (DMSO) were used as the controls. After 72 hours, cells were washed with PBS and replenished with fresh normal culture medium. Cells were maintained in culture for 2–5 weeks, with medium change twice a week [29], [31]. The scoring of foci was performed according to the established guidelines [28], [31], [32]. Only foci classified as type III which featured characteristics such as basophilic, dense multilayered, cells randomly orientated at the focus edge, invasion into the monolayer, and spindle-shaped, were scored as positive and counted. Foci of less than 1 mm in diameter were not analyzed. The transformation frequency was calculated only by dividing the total number of type III foci with the number of seeding cells. In the morphological analysis of individual cell, a cell that featured a high number of stress fibers and a nonpolarized cell shape was designated as state 1; cells that exhibited a low stress content and an elongated shape were classified as state 2 [33].

### Immunofluorescence

Cells cultured in the indicated experimental conditions were harvested and fixed with 4% v/v paraformaldehyde (Sigma-Aldrich, St. Louis, MO, USA). Cells were pretreated with 0.1% Triton-X-100 (Sigma-Aldrich, St. Louis, MO, USA) and 0.05% SDS in PBS, followed by 0.2% BSA/0.1% saponin/PBS for 30 minutes. Fixed cells were stained with the anti-actin antibody (1∶200; Sigma-Aldrich, St. Louis, MO, USA) and were subsequently stained with rhodamine-phalloidin (1∶40; Invitrogen, Carlsbad, CA, USA) for actin visualization [34], [35]. Nuclei were stained with DAPI (1∶5000). Fluorescence was photographed and merged using a confocal microscope (Leica SP-5).

### Biological assays

For the scratch wound assay, cells were cultured as confluent monolayers and were wounded by removing a 1 cm strip of cells across the well [36]. A phase-contrast microscope equipped with a culture chamber (Zeiss Axiovert 200) was used to record the cell migration in a time-lapse manner. The results were analyzed using MetaMorph software (Universal Imaging Corporation, West Chester, PA). For the proliferation assays, cells were seeded in a 96-well plate (1×10^4^ per well). After 24 hours culture, cells were pulsed with 5-bromo-2′-deoxyuridine (BrdU). Subsequently, the incorporation rate of BrdU was determined using an enzyme-linked immunosorbent assay kit (Roche, Diagnostics, IN, US). For a terminal transferase uridyl nick end labeling (TUNEL) assay, an *in situ* cell death detection kit (Roche, Diagnostics, Indianapolis, IN) was used to detect apoptosis with flow cytometry. For anchorage-independent growth assays, the ability of colony formation of untreated and MCA-treated BALB/c 3T3 cells was tested in a semi-solidified agarose gel. The lower gel consisted of 1.5% agarose and the top gel consisted of 0.4% low melt agarose. After 2 weeks of incubation, MTT was used for cell staining. Those cells with positive MTT reactions were photographed and analyzed. Quantification was performed when the colony-forming efficiency was calculated by dividing the number of colonies by the number of seeding cells. For the analysis of the *in vitro* invasion, the invasive potential of cells was measured in an 8-μm pore transwell whose top inserts were pre-coated with collagen gel (Coastar). The lower inserts were filled with 0.7 ml of DMEM medium containing 10% FBS. The number of invasive cells on the lower surface of the membrane was counted. All experiments were repeated at least three times.

### Flow Cytometry

Cells growing on 6 cm culture dishes were treated with 2 μM BMVC for 15 min. The cells were then collected by trypsinization and washed with PBS. Samples were analyzed using flow cytometry (FACS Caliber and CellQuest software, BD Biosciences). Ten thousand cells were analyzed at each condition with a blue argon laser (488-nm wavelength at 500 mW). The gating threshold was adjusted for particles that were smaller than cells and was read with a logarithmic amplification of the parameters. All experiments were repeated at least three times.

### Statistical analysis

For the statistical comparison of values in the different experiments of transformed cells and controls, a Student's t*-*test and an ANOVA test were used. For the correlation between BMVC expression and the degree of cell transformation, the bivariate correlation was performed and the Pearson's correlation coefficient was determined. For each experiment, the respective *p* value was calculated and a statistically significant result was considered when the *p* value was less than 0.05.

## Results

### Induction of cell transformation

**Figure 1:**
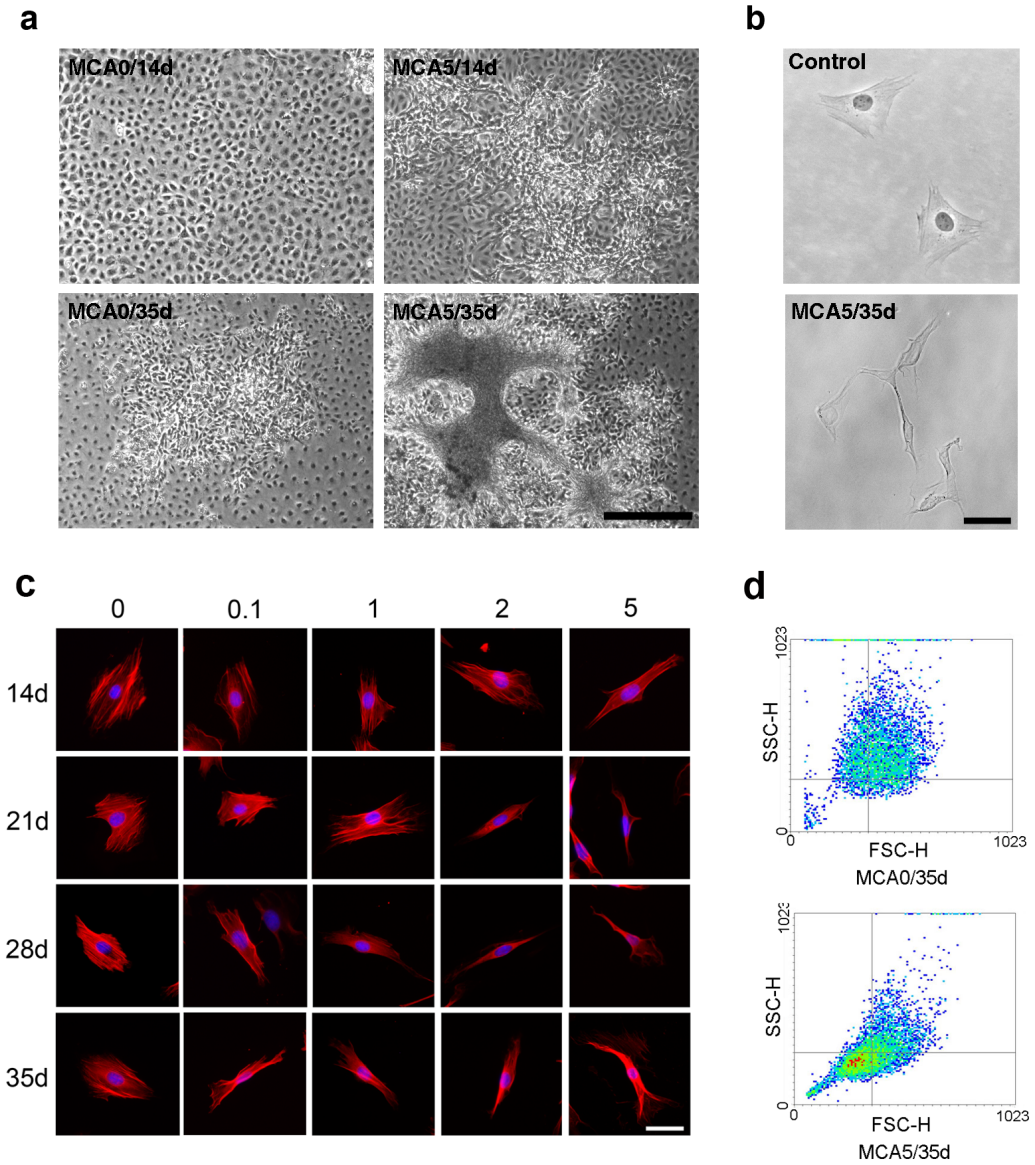
MCA induced cell transformation of BALB/c 3T3 cells. (a) Representative microscopic appearance of MCA-induced foci. BALB/c 3T3 cells were treated with 5 μg/ml MCA (MCA5) and vehicle (MCA0) separately, and cultured for 14 (14d) and 35 days (35d); scale bar  = 500 μm. (b) Cell morphology of individual cells of untreated BALB/c 3T3 cells (control) and MCA-treated BALB/c 3T3 cells (MCA5/35d); scale bar  = 50 μm. (c) Alteration of morphological phenotypes and cytoskeleton organization of BALB/c 3T3 cells treated by different MCA doses (0.1, 1, 2, 5 μg/ml) and cultured periods (14, 21, 28, 35 days); smooth muscle actin (red), DAPI (blue), scale bar  = 50 μm. (d) Cells in the control and MCA-treated groups were analyzed using flow cytometry. The forward scatter (FSC) and side scatter (SSC) of particles were simultaneously measured. All experiments had been repeated at least three times.

In this study, a well-recognized system using mutagen-treated BALB/c 3T3 cells was established [31], [37]. After MCA treatment, BALB/c 3T3 cells started to form foci after the indicated culture periods (Figure 1a). Cells treated by MCA were dense, multilayered, and randomly oriented. The morphology of cell became elongated and spindle (Figure 1b) [38]. In subcellular level, BALB/c 3T3 cells usually exhibited numerous stress fibers and nonpolarized cell shapes. The number of stress fibers decreased in the MCA-treated cells (Figure 1c). Quantitatively, all cells in the untreated group belonged to state 1 regardless of the culture period. Nevertheless, the MCA-treated cells were classified as state 2, especially after long culture periods. In the cytometric analysis, the transformed cells typically demonstrated smaller and elliptical shapes (Figure 1d). There results indicate that MCA treatment significantly affects the original morphology and the cytoskeleton arrangements of cells.

### BMVC expression in MCA-treated cell

**Figure 2:**
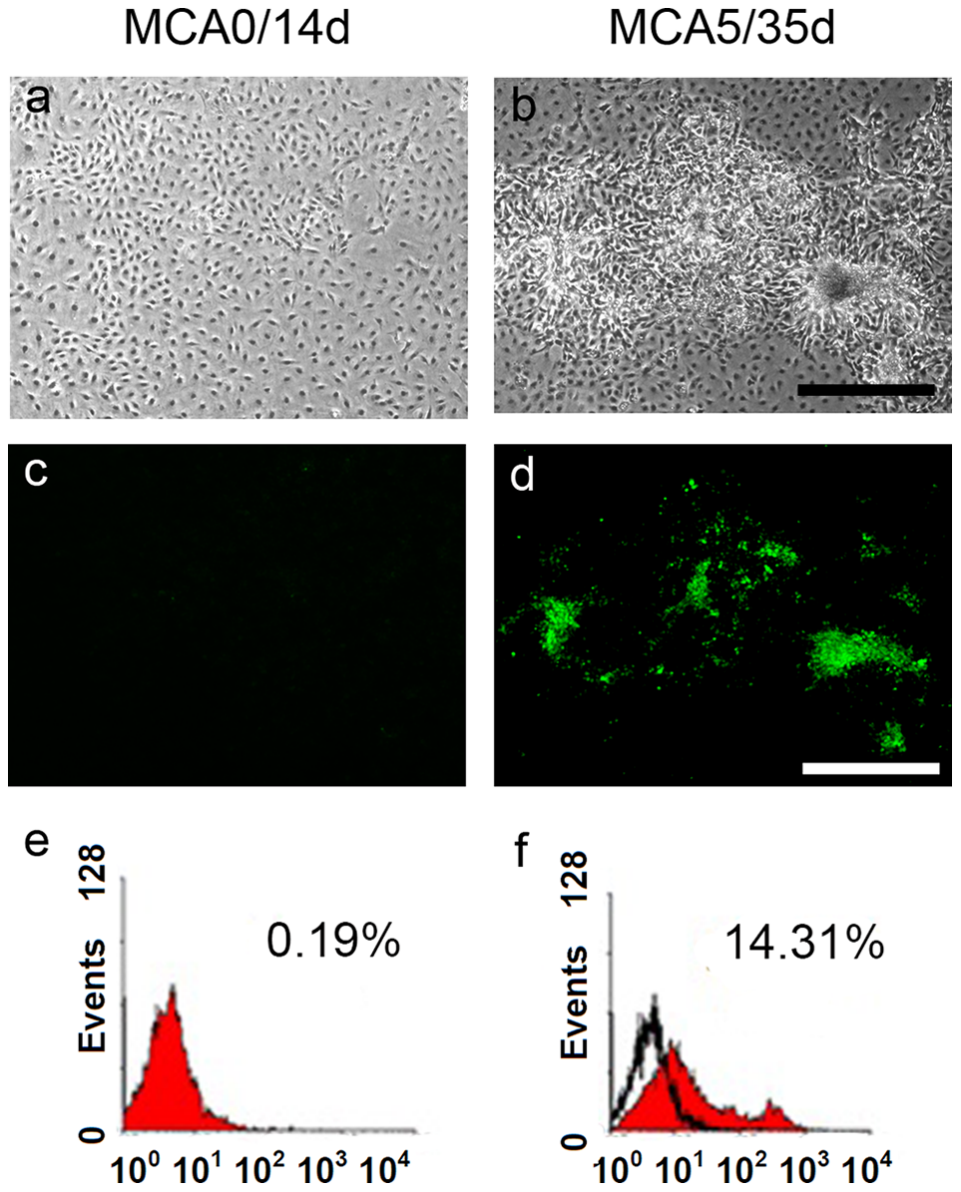
BMVC expression of transformed cells in a time- and dose- dependent manner. (a, b) Morphological phenotypes of BLAB/c 3T3 cells treated with MCA of different doses (0, 5 μg/ml) and cultured for different periods (14, 35 days). Scale bar  = 500 μm. (c, d) BMVC expressions of MCA-treated cells were detected by fluorescence microscopy. Scale bar  = 500 μm. (e, f) The percentage of positive BMVC expression of MCA-treated cells was determined and analyzed by flow cytometry.

In our CTA system, the number of foci formation increased with an increased MCA concentration and a long culture period, showing a dose- and time-dependent manner (Figure 2a, Figure S1a). The cells from each group may harbor a varied degree of cell transformation, and could be used to verify the capacity of BMVC probes (Figure 2b). After 21-day culture, BMVC expression was only detectable in MCA2 and MCA5 groups. After 35-day culture, BMVC fluorescence was noted in MCA1, MCA2, and MCA5 groups (). The cytometric data showed that the number of BMVC-positive cells increased in the group that was treated with a high dose of MCA and cultured for a long period (Figure 2c, Figure S1c). These results indicate that the number of BMVC-positive cells increases in accordance with the dose and the duration of MCA treatment.

Additionally, to further confirm that the phenomenon of BMVC expression was not only exclusively observed in an MCA-induced BALB/c 3T3 cell model, the cells from different species and tissue were induced with distinct carcinogens. It could confirm that BMVC probes were able to be generally applied to other transformed cells. Hs-68 cells, the human fibroblast cells, were treated with ultraviolet light (UV) and irradiation separately [39], [40]. In the UV system, the BMVC fraction increased with the light intensity and increased in accordance with the culture periods (Figure S2). Similar results were found in the groups that were treated with γ-rays (Figure S3). These results confirmed that BMVC probes could be applied to other different types of transformed cells induced by distinct carcinogenic agents.

### BMVC capacity of detecting foci formation during carcinogenesis

**Figure 3:**
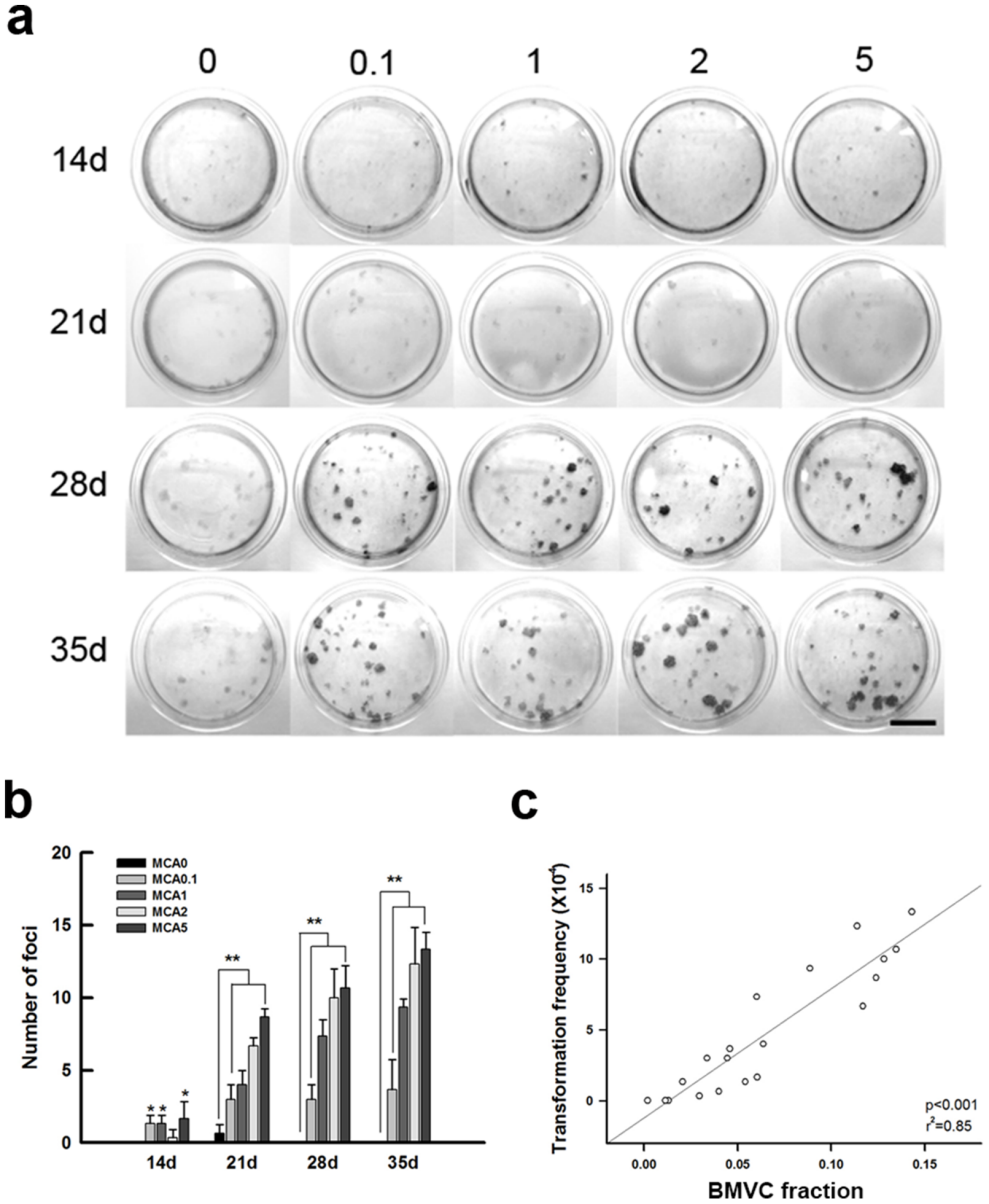
The correlation between the BMVC fraction and the frequency of foci formation in transformed cells. (a) Foci formation of BALB/c 3T3 cells induced by different MCA doses (0.1, 1, 2, 5 μg/ml) and cultured periods (14, 21, 28, 35 days); scale bar  = 2 cm. (b) Histogram representing the quantification of foci formation. (*p<0.05; **p<0.001 compared to the matched non-treated cells, paired t-test). (c) The correlation between the BMVC fraction and the frequency of foci formation expressed as the transformation frequency.

To serve as a useful marker for early detection of cancer formation, BMVC probes are required to recognize different features of transforming cells. The formation of foci is an important hallmark of carcinogenesis [41], [42]. Thus, the model showing distinct frequency of foci formation was established [28], [31], [32]. Foci appeared in the groups that were treated with high MCA doses and long culture periods, and the foci increased not only in size but also in numbers (Figure 3a). Quantitatively, the increase of foci number was initially identified after 14 days and was more obvious thereafter (Figure 3b). A dose and time dependent manner was observed. When BMVC probes were applied, the BMVC fraction significantly correlated with the frequency of foci formation (Figure 3c). The results indicate that BMVC probes were able to detect the foci formation during cell transformation.

### BMVC capacity of detecting cell motility during carcinogenesis

**Figure 4:**
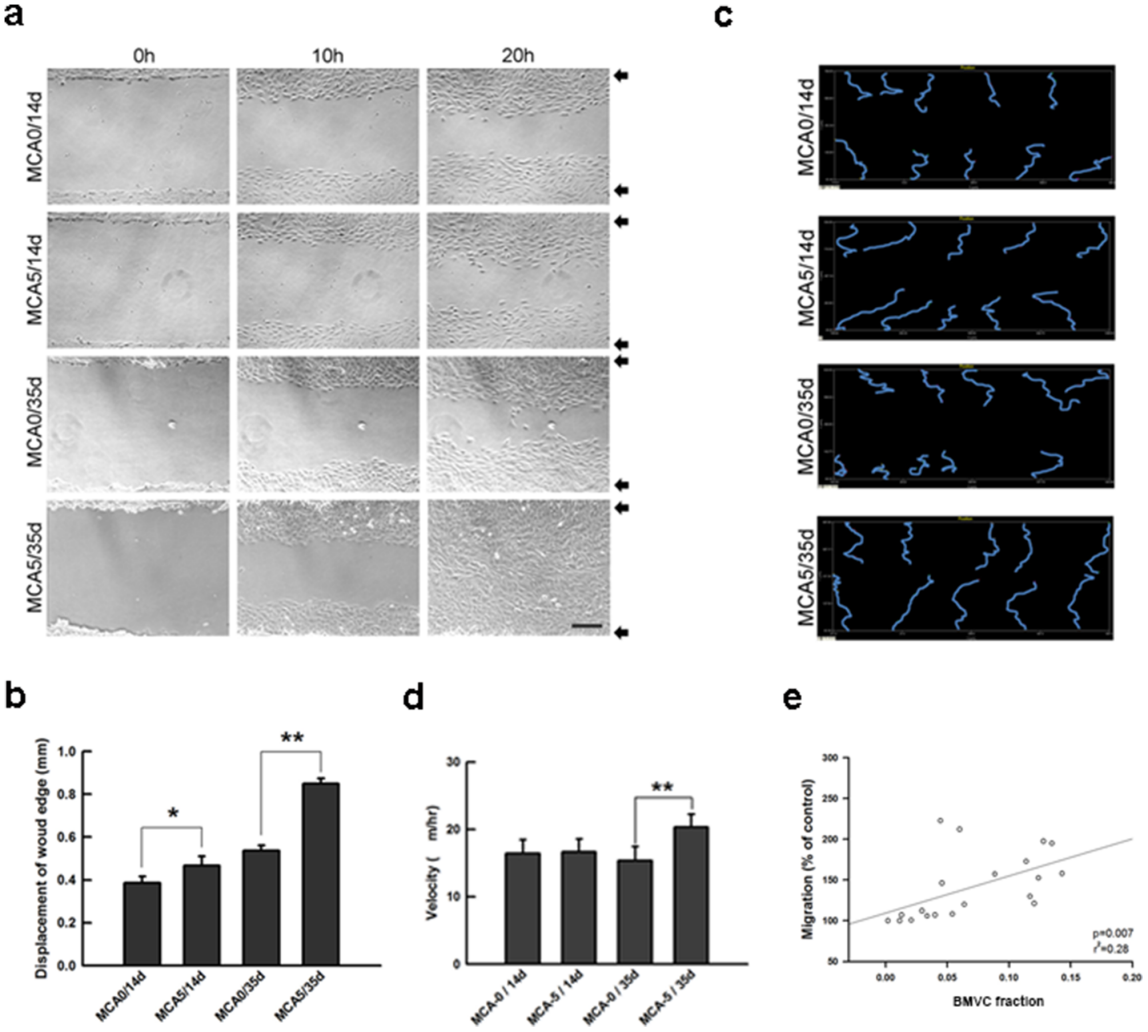
The correlation between the BMVC fraction and cell motility of the transformed cells. (a) A scratch wound assay was used to detect cell migration of BALB/c 3T3 cells from different experimental groups. Scale bar  = 500 μm; Arrows: the wound edge of 0 h. (b) Quantification of the results of the scratch wound assay. The cell movement was presented as the displacement of the wound age (*p<0.05; **p<0.001, t-test). (c) The migratory path of a single cell was traced from the point of the original location in a time-lapse manner. (d) Quantitative analysis of a single cell migration is presented as the migratory velocity (**P<0.001, t-test). (e) The correlation between the BMVC fraction and the cell motility of MCA-treated cells.

Increased cell motility is also a hallmark of cell transformation during carcinogenesis. To test the BMVC capacity of detecting this feature of transforming cells, a scrape wound assay was established. In the MCA5/35d group, the wound was completely repaired after 20 hours (Figure 4a). In the cells that were cultured for long periods, the ability of wound repair significantly increased (Figure 4b). A time-lapse analysis demonstrated that most tracks of the control group were jagged lines, presenting the random motion of migratory cells. In contrast, most of the migratory tracks of MCA-treated cells were straight (Figure 4c). The results further confirmed the migratory ability of MCA-treated cells [43]. Quantification analysis showed that the cell migratory ability was significantly enhanced in the MCA5/35d group (Figure 4d). When BMVC probes were applied, a significant correlation was found between the BMVC fraction and the migratory ability of transformed cells (Figure 4e), showing the capacity of BMVC probes to detect the increased cell motility of transformed cells.

### BMVC capacity of monitoring cell proliferation and apoptosis during carcinogenesis

**Figure 5:**
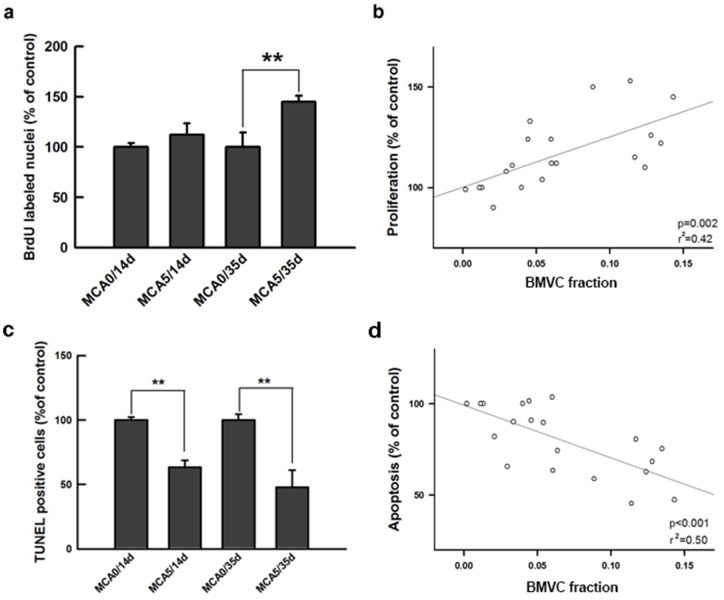
BMVC expression correlates with proliferation and apoptosis of transformed cells. (a) Cell proliferation was tested by measuring the uptake of BrdU. (**P<0.001, t-test). (b) The correlation between the BMVC fraction and cell proliferation of the MCA-treated cells. (c) Apoptosis was tested by measuring the TUNEL-positive cells in the indicated experiment groups. (**P<0.001, t-test). (d) The correlation between the BMVC fraction and the cell apoptosis of MCA-treated cells.

Unlimited cell proliferation is an important characteristic of cell transformation during carcinogenesis [3], [4], [38], [44]. In this study, cell proliferation was determined by BrdU incorporation. After 35-day culture, the percentage of BrdU positive cells was significantly greater in the MCA-treated group compared to untreated cells (Figure 5a). When applying BMVC probes, the BMVC fraction significantly correlated with the proliferative ability of cells (Figure 5b). Decreased apoptosis is another important feature of transformed cells [3], [4]. It was found that the percentage of TUNEL-positive cells decreased in the MCA-treated group after a 14-day culture (Figure 5c). The proportion of BMVC-positive cells significantly correlated with the percentage of cell apoptosis in a negative trend (Figure 5d). This observation indicates that the ability to evade apoptosis is greater in the BMVC-positive cells.

### BMVC capacity of detecting anchorage independent cell growth during carcinogenesis

**Figure 6:**
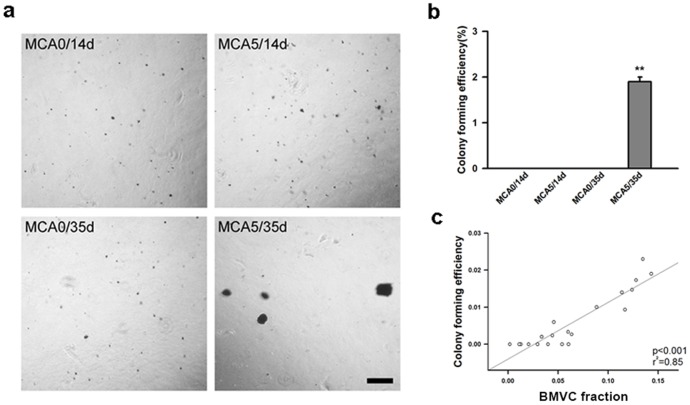
The correlation between the BMVC fraction and anchorage independent growth of the transformed cells. (a) A soft agar assay was used to detect the anchorage independent growth of transformed cells from different experimental groups. Scale bar  = 500 μm. (b) The quantitative analysis of the soft agar assay. The colony forming efficiency was calculated and compared (**P<0.001, t-test). (c) The correlation between the BMVC fraction and colony forming efficiency of transformed cells in the soft agar.

Anchorage-independent growth is another feature of cell malignant transformation [3], [4]. Cultured in soft agar, cells of the untreated groups were unable to grow, even after a long culture period. However, MCA-treated cells continued to grow and developed colonies (Figure 6a). The quantitative results confirmed the properties of anchorage independent growth (Figure 6b). When the cells were stained with BMVC probes, the BMVC fraction significantly correlated with colony-forming efficiency (Figure 6c). It demonstrated the potential of BMVC probes to detect anchorage independent growth of transformed cells.

### BMVC capacity of detecting cell invasiveness during carcinogenesis

**Figure 7:**
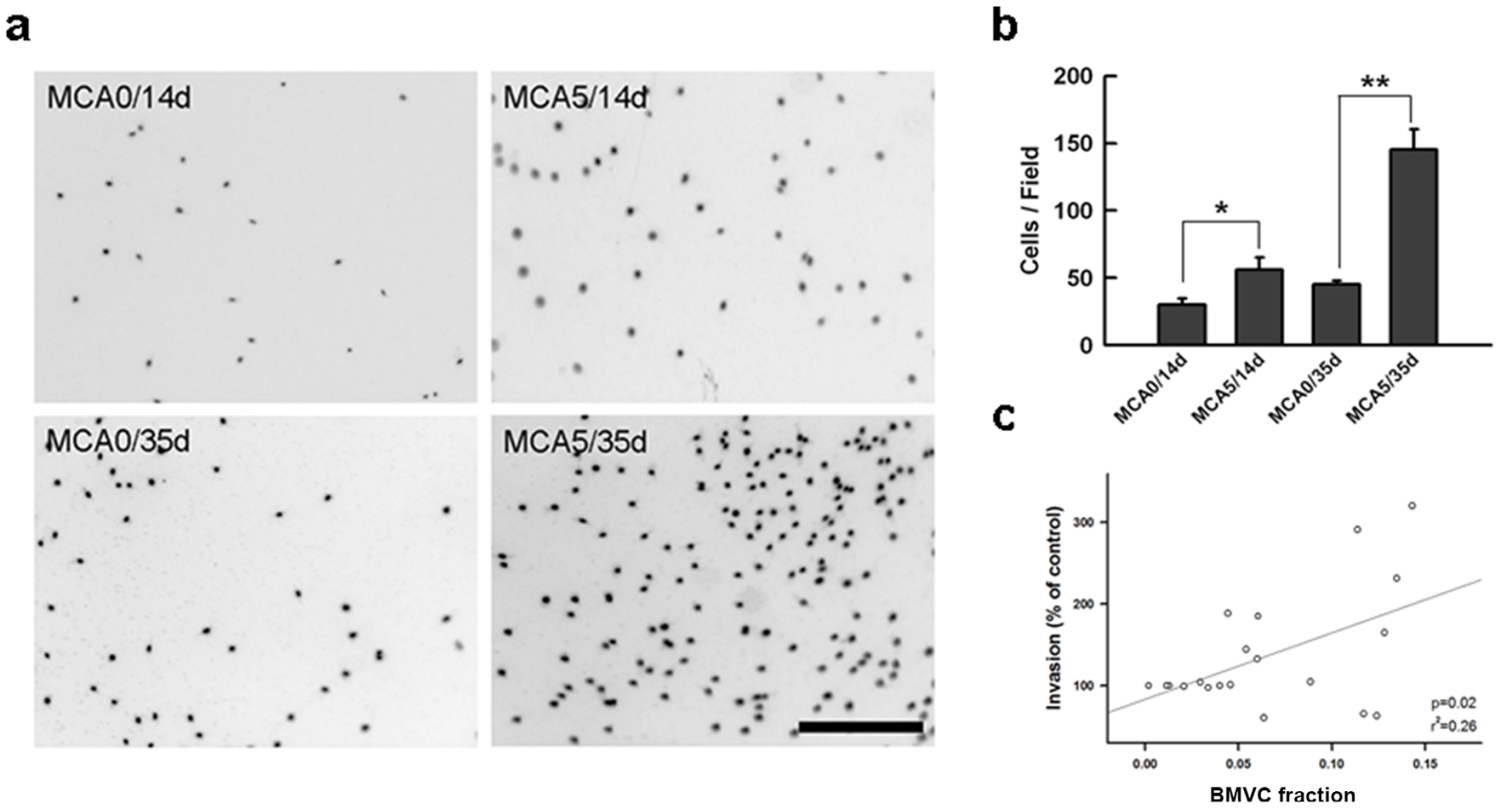
The correlation between the BMVC fraction and the invasive ability of transformed cells. (a) The transwell assay was used to detect the invasive ability of transformed cells from different experimental groups. Scale bar  = 500 μm. (b) The quantitative analysis of the transwell assay. The number of invading cells were calculated and compared (**P<0.001, t-test). (c) The correlation between BMVC fraction and the invasiveness of MCA-treated cells.

The ability of invasion is mandatory for cells to be malignantly transformed and metastasize [3], [4]. After 14-day culture, MCA-treated cells started to demonstrate invasive characteristics (Figure 7a) [45]. The number of cells that successfully penetrated the extracellular matrix was greater than that of the control (Figure 7b). In 35-day culture, more cells were observed in the transwell chamber of MCA-treated group than untreated cells (Figure 7b). The BMVC expression significantly correlated with the proportion of invading cells (Figure 7c). The potential of BMVC probes to detect the invasiveness of transformed cells was confirmed by the results.

### Significance of BMVC capacity in detecting the features of transformed cells

According to the results mentioned above, BMVC was able to detect many transformed cell features during carcinogenesis (Table 1). The Pearson product moment correlation coefficient and the statistical significance of each characteristic were listed (Table 2). These results provide evidence that BMVC could serve as a useful marker for early detection of cancer formation.

**Table 1:** Comparison of the cellular features of the untreated cells (MCA0) and the cells treated with MCA (MCA5) for 35 days.

Cellular features	Untreated cells (MCA0)	MCA-treated cells (MCA5)	*p* value
Transformation Frequency (No. of type III foci)	13.33±1.04	0.00±0.00	<0.001
Migration (mm)	0.54±0.02	0.85±0.31	<0.001
Velocity (µm/hour)	20.53±2.11	15.79±2.11	<0.001
Proliferation (BrdU labeled nuclei, % of untreated cells)	100.00±15	147.83±6.43	<0.001
Apoptosis (TUNEL positive cells, % of untreated cells)	100.00±4.69	93.75±13.28	<0.001
Colony Forming Efficiency (%)	0.00±0.00	1.94±0.10	<0.001
Invasion (cells/field)	46.51±2.33	144.19±13.95	<0.001

**Table 2:** Significance of the correlation between cellular characteristics and the BMVC fraction in transformed cells.

Characteristics of transformed cell	Pearson product moment correlation coefficient	p value
Transformation Frequency	0.92	8.86 E-09
Migration	0.65	6.97 E-03
Proliferation	0.58	2.11 E-03
Apoptosis	−0.71	4.98 E-04
Colony Forming Efficiency	0.92	7.87 E-09
Invasion	0.51	2.11 E-02

## Discussion

In the current study, BMVC was capable of detecting distinct cellular features and behaviors during carcinogenic transformation. In addition to the understanding that BMVC can be used as a marker for cancer detection [20], [21], our current study extends the scope of BMVC application from cancer cells to cells whose transformation is ongoing. It demonstrates the potential of BMVC probe of identifying pre-cancerous lesions for early cancer detection.

During the carcinogenic process of cellular transformation, gaining the ability of unlimited proliferation is important [2]–[4]. Because telomere maintenance is essential for unlimited proliferation, it becomes a hallmark of transformed cells. Higher expression of telomerase has been found in most human tumors [46], [47]. These features suggest that telomere and telomerase are good targets for cancer detection and treatment. During carcinogenesis, up-regulation of telomerase, which is critical to stabilize the telomere, is correlated with tumor progression, invasion, and metastasis [48]. Telomere has a G-rich single-stranded tail that can bind together to generate an intramolecular G-quadruplex structure [49], [50]. Many molecules that can interact with G-quadruplexes are widely used in cancer researches, and are regarded as potential markers for cancer detection and therapy [51]–[56]. BMVC has a preferential binding to the G-quadruplex structure of DNA, and its intensity of fluorescence increases during binding reactions [17]–[19].

In this study, BMVC probes offer a convenient color discrimination method for detecting cell transformation. The alterations of BMVC expression correlate with the degrees of cell transformation. By demonstrating its capacity in several cell lines and different CTA systems, BMVC probes are effective in detecting cell transformation during carcinogenesis. Although many probes have been used to detect cell transformation, few exhibited the correlation between specific cellular behaviors and responses during carcinogenic transformation. Our results provide evidence that BMVC can be used to monitor specific characteristics of transforming cells, which is important for the early detection of carcinogenesis. BMVC probes are capable of identifying the changes of morphology, function, and behavior of transforming cells. The fluorescent color is only expressed in the transforming cells rather than the other non-transforming ones. Unlike the other staining methods, the features of BMVC probes render the color discrimination easier to be distinguished [57].

Before applying BMVC probes in animal studies and clinical trials, CTA is a good test for verifying BMVC capacity. The CTA has been well recognized as a unique system that is competent for identifying the potential carcinogen and is also regarded as the only possible *in vitro* alternative to the carcinogenetic studies that are conducted in animals [42], [58], [59]. This assay is based on the malignant transformation of BALB/c 3T3 cells, which includes the initiating and promoting stages of carcinogenesis [57]. It has been shown that CTA closely models the different stages of carcinogenesis *in vivo*, including both cellular and molecular alterations. In addition, the CTA has been acknowledged as relevant to carcinogenesis with established reliability and reproducibility [42], [58], [60]. In this study, the development of cell abilities including migration, proliferation, evading of apoptosis, anchorage independent growth, and invasion were demonstrated. In this system, the degree of cell transformation can be induced in a dose- and time-dependent manner, which makes it possible to fine tune the process of cell transformation and render it an appropriate model for investigating the capacity of BMVC probes.

When normal cells are progressively transformed to become neoplastic, they acquire a succession of capacities, such as self-sufficiency in proliferative signaling, insensitivity to growth suppressors, limitless replicative potential, resistance to cell death, and potential of invasion and metastasis.[3], [4] It is well acknowledged that carcinogenesis is a multistep process, and each step is essential to enable a cell to become tumorigenic or carcinogenic. These alterations of cell phenotypes, behaviors, and physiological responses collectively dictate the successful evasion of the regulation of normal cells, and comprehensively substantiate the understanding of cancer biology. Because there are intermediate states through which normal cells evolve progressively into cancer, the probes that are used for early cancer detection should target the evolving status. In our results, BMVC expression significantly correlated with many important hallmarks of cell transformation, including transformation frequency, migratory ability, cell proliferation, evasion of apoptosis, colony forming efficiency, and tumor invasive capacity. It indicates that the BMVC probe is competent to be a sensitive and specific detector of cell transformation.

Among different characteristics of cell transformation, the statistic of BMVC expression is the most significant in the colony formation ability. Contact inhibition is abolished in transformed cells, which allows sustained cell proliferation and finally results in the formation of tumor colonies [3], [4]. Likewise, anchorage dependence is also a barrier to prevent normal cells from neoplastic transformation [61]. When anchorage independence occurs, the transformed cells are able to grow without surface attachment and loosen the connection with neighboring extracellular matrix, which is an important characteristic of tumorigenesis. In the BALB/c 3T3 model, a subset of anchorage independent genes have been identified in the expression profiles of transformed cells [62]. On the other hand, the ability of transformed cells to evade apoptosis is also significantly associated with BMVC expression. The acquisition of the ability to resist apoptosis is imperative for cells to be transformed [3], [4], [63]. Accordingly, a strong association between the specific features of transformed cells with BMVC expression substantiates the potential applications of BMVC probes.

## Conclusion

The BMVC probe can identify cell transformation during carcinogenesis. Distinguished fluorescent color difference is detected in transformed cell with specificity. BMVC signals significantly correlate with specific hallmarks of transforming cells. Our results demonstrated the competence of BMVC probes to detect cell transformation, suggesting BMVC probes are of great potentials to be used to detect pre-cancerous lesions for early cancer detection.

## Supporting Information

Figure S1 **BMVC expression of transformed cells in a time- and dose- dependent manner.**
(PDF)

Figure S2 **Increased expression of BMVC in UV-treated cells.**
(PDF)

Figure S3 **Increased expression of BMVC in γ-ray treated cells.**
(PDF)

Materials and Methods S1 (DOCX)
