# Supplementary material for: Detection of Cell Carcinogenic Transformation by a Quadruplex DNA Binding Fluorescent Probe
Source: PLoS One. 2014 Jan 28;9(1):e86143. doi: 10.1371/journal.pone.0086143 (PMC3904876; doi:10.1371/journal.pone.0086143)
Supplement: Figure S1 — BMVC expression of transformed cells in a time- and dose- dependent manner. (PDF) [file pone.0086143.s001.pdf]

Figure S1. BMVC expression of transformed cells in a time- and dose- dependent manner.

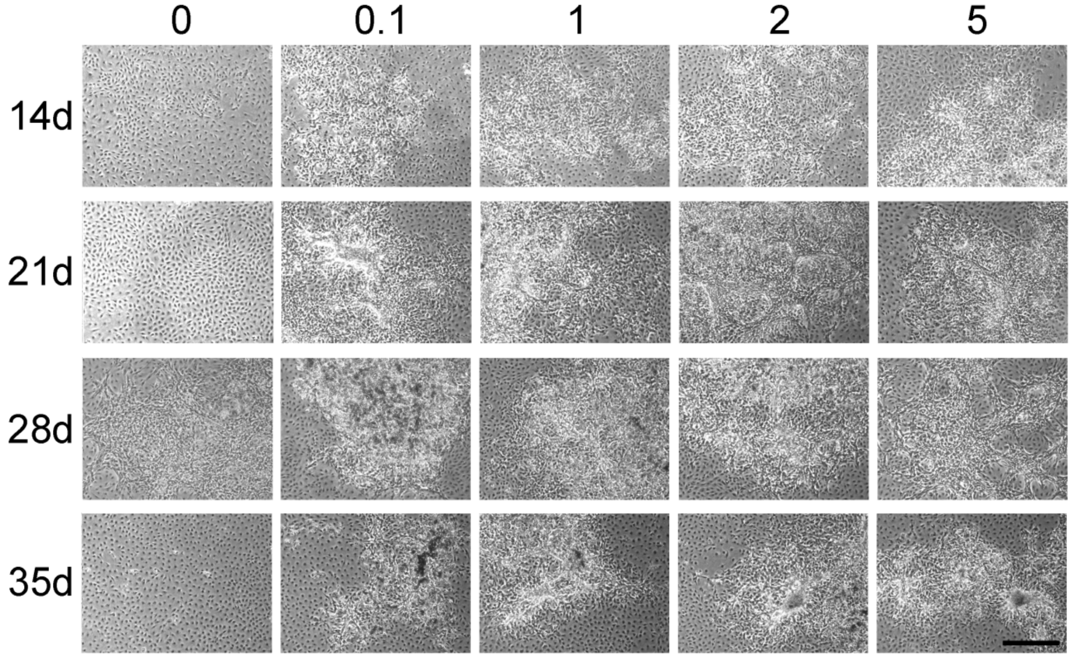

(a) Morphological phenotypes of BLAB/c 3T3 cells treated with MCA of different doses (0, 0.1, 1, 2, 5 μg/ml) and cultured for different periods (14, 21, 28, 35 days); scale bar = 500 μm.

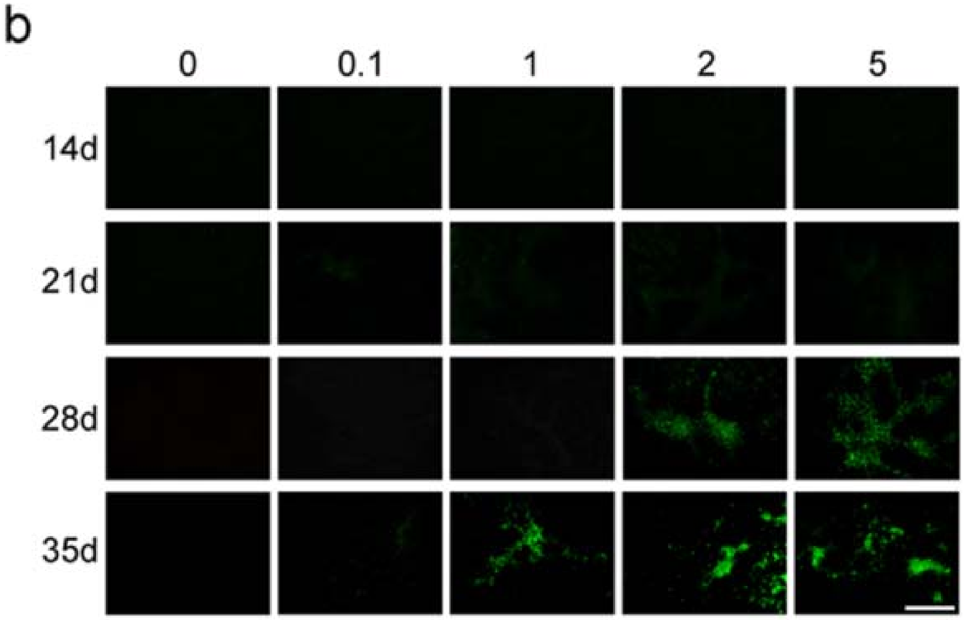

(b) BMVC expressions of MCA-treated cells were detected by fluorescence microscopy; scale bar = 500 µm.

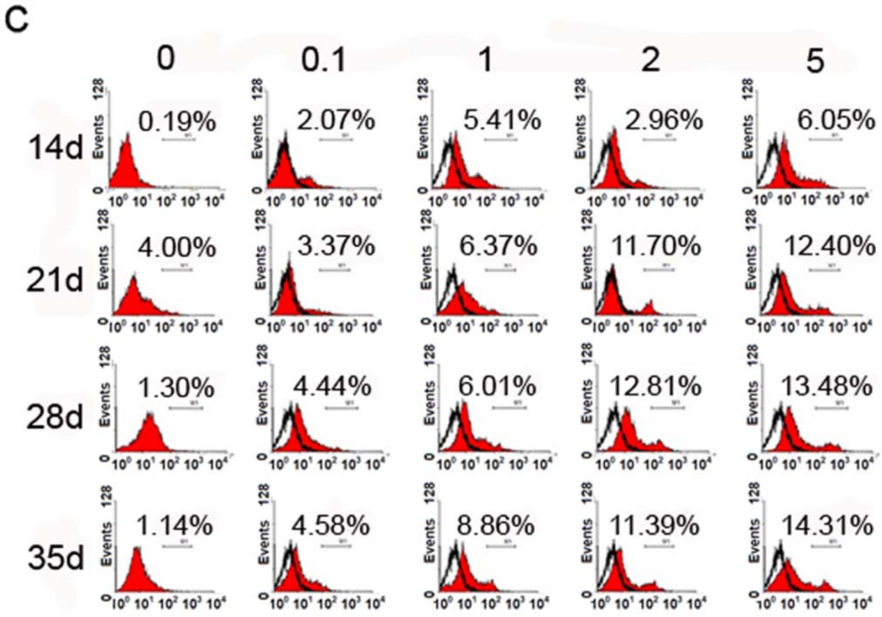

(c) The percentage of positive BMVC expression of MCA-treated cells was determined and analyzed by flow cytometry
