# Supplementary material for: Detection of Cell Carcinogenic Transformation by a Quadruplex DNA Binding Fluorescent Probe
Source: PLoS One. 2014 Jan 28;9(1):e86143. doi: 10.1371/journal.pone.0086143 (PMC3904876; doi:10.1371/journal.pone.0086143)

Figure S2

Increased expression of BMVC in UV-treated cells. Cultured cells were treated with 0, 0.69, 1.38, 2.07, 2.76J UV for (a)0, (b)1, (c)3, (d)5, (e)7, (f)9 days.

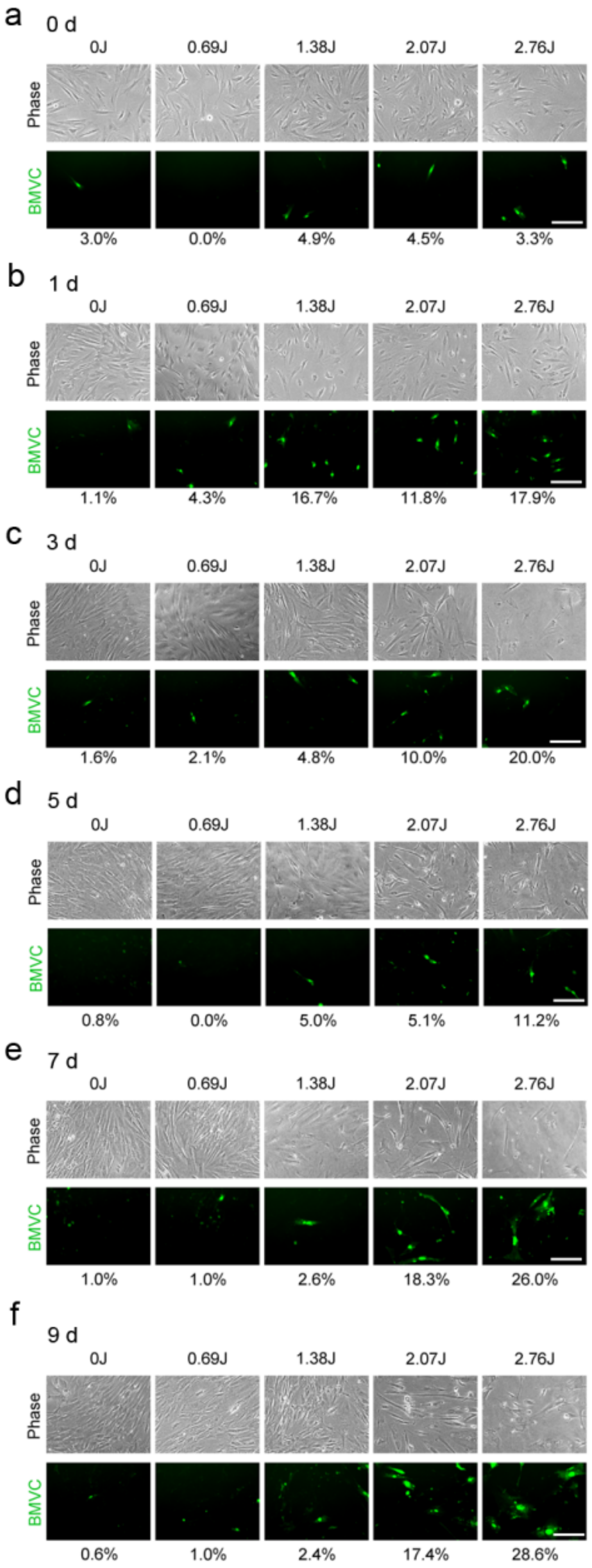

Supplement: Figure S2 — Increased expression of BMVC in UV-treated cells. (PDF) [file pone.0086143.s002.pdf]
