# Supplementary material for: Detection of Cell Carcinogenic Transformation by a Quadruplex DNA Binding Fluorescent Probe
Source: PLoS One. 2014 Jan 28;9(1):e86143. doi: 10.1371/journal.pone.0086143 (PMC3904876; doi:10.1371/journal.pone.0086143)

Figure S3

Increased expression of BMVC in γ-ray treated cells. Cultured cells were treated with 0, 0.5, 1, 1.5, 2 Gy γ-ray for (a)0 and (b)1 day.

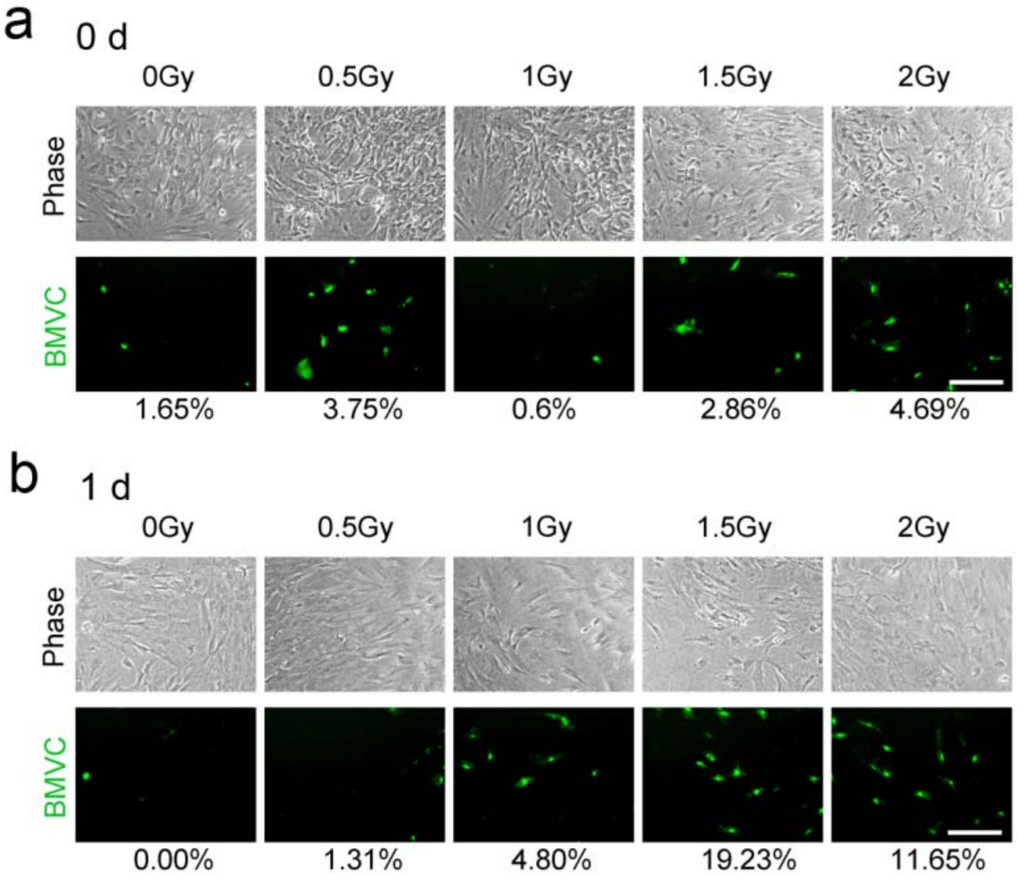

Supplement: Figure S3 — Increased expression of BMVC in γ-ray treated cells. (PDF) [file pone.0086143.s003.pdf]
