# Supplementary material for: Detection of Cell Carcinogenic Transformation by a Quadruplex DNA Binding Fluorescent Probe
Source: PLoS One. 2014 Jan 28;9(1):e86143. doi: 10.1371/journal.pone.0086143 (PMC3904876; doi:10.1371/journal.pone.0086143)
Supplement: Materials and Methods S1 — (DOCX) [file pone.0086143.s004.docx]

**Supplementary Information**

The human fibroblast cell lines (Hs-68) were obtained from the American Type Culture Collection (ATCC). Cell culture was performed based on the protocol suggested by ATCC, and maintained in an incubator with 37°C, 5%CO_2_, and 95% humidity. UV irradiation was performed at room temperature in an irradiator containing 6 x 8 w UV-B fluorescent tubes with a Laser Power Meter, and a shutter to minimize the inequalities of doses at the edges of the culture dishes. Over 90% of the UV irradiation was emitted from these lamps at a wavelength of 312 nm. The dose rate to the cells was 0.023 J/sq m sec. The γ-radiation source used was a 137 Cs self-shielded irradiator. The dose rate employed was 0.662 MeV. The overlying culture medium was removed prior to UV irradiation and was replaced with fresh medium immediately thereafter.
